# Co-expression Gene Network Analysis and Functional Module Identification in Bamboo Growth and Development

**DOI:** 10.3389/fgene.2018.00574

**Published:** 2018-11-27

**Authors:** Xuelian Ma, Hansheng Zhao, Wenying Xu, Qi You, Hengyu Yan, Zhimin Gao, Zhen Su

**Affiliations:** ^1^State Key Laboratory of Plant Physiology and Biochemistry, College of Biological Sciences, China Agricultural University, Beijing, China; ^2^State Forestry Administration Key Open Laboratory on the Science and Technology of Bamboo and Rattan, Institute of Gene Science for Bamboo and Rattan Resources, International Center for Bamboo and Rattan, Beijing, China

**Keywords:** bamboo, gene network analysis, functional module, gene expression views, growth and development

## Abstract

Bamboo is one of the fastest-growing non-timber forest plants. Moso bamboo (*Phyllostachys edulis*) is the most economically valuable bamboo in Asia, especially in China. With the release of the whole-genome sequence of moso bamboo, there are increasing demands for refined annotation of bamboo genes. Recently, large amounts of bamboo transcriptome data have become available, including data on the multiple growth stages of tissues. It is now feasible for us to construct co-expression networks to improve bamboo gene annotation and reveal the relationships between gene expression and growth traits. We integrated the genome sequence of moso bamboo and 78 transcriptome data sets to build genome-wide global and conditional co-expression networks. We overlaid the gene expression results onto the network with multiple dimensions (different development stages). Through combining the co-expression network, module classification and function enrichment tools, we identified 1,896 functional modules related to bamboo development, which covered functions such as photosynthesis, hormone biosynthesis, signal transduction, and secondary cell wall biosynthesis. Furthermore, an online database (http://bioinformatics.cau.edu.cn/bamboo) was built for searching the moso bamboo co-expression network and module enrichment analysis. Our database also includes *cis*-element analysis, gene set enrichment analysis, and other tools. In summary, we integrated public and in-house bamboo transcriptome data sets and carried out co-expression network analysis and functional module identification. Through data mining, we have yielded some novel insights into the regulation of growth and development. Our established online database might be convenient for the bamboo research community to identify functional genes or modules with important traits.

## Introduction

Bamboo, an important fast-growing non-timber forest plant worldwide, has been an essential forest resource with an annual trade value of >2.5 billion US dollars, and approximately 2.5 billion people depend on it economically (Peng et al., 2013a,b; Zhao et al., 2017). Moso bamboo (*Phyllostachys edulis*, once known as *Phyllostachys heterocycla*) is the most economically valuable bamboo in Asia, especially in China. With the release of the whole-genome sequence of moso bamboo, there are increasing demands for refined annotation of bamboo genes on the whole-genome level. Considering the small proportion of annotated genes in the bamboo genome and high accumulation of data, it is necessary and urgent to conduct big-data mining to yield novel insights into bamboo growth and development.

Generally, genes with coordinated expression across a variety of experimental conditions indicate the presence of functional linkages between genes. Thus, co-expression gene networks can associate these genes of unknown function with biological processes in an intuitive way. An increasing number of studies have supported the versatility of co-expression analysis for inferring and annotating gene functions (D’Haeseleer et al., 2000; Aoki et al., 2007; Usadel et al., 2009; Morenorisueno et al., 2010; Li et al., 2015; Serin et al., 2016). Through data mining tools and algorithms that describe complex co-expression patterns of multiple genes in a pairwise fashion, global co-expression network analyses consider all samples (multiple data sources with independence) together and establish connections between genes based on the collective information available (Bassel et al., 2011). Compared with such a network, the conditional co-expression network aims to enhance our understanding of gene function from a portion of transcriptome data sets that have much in common, such as having the same source and a similar acquisition of raw materials and inferring gene transcriptional regulatory mechanisms in developmental processes based on a series of selected associated samples. In co-expression analysis, gene expression views can help clearly present the tendency of differential gene expression between samples. Consequently, co-expression networks with expression views can be used to associate genes of unknown function with biological processes, to discern gene transcriptional regulatory mechanisms *in vivo* and to prioritize candidate regulatory genes or modules of vital traits.

Based on the de novo sequencing data, together with the full-length complementary DNA and RNA-seq data of moso bamboo, BambooGDB has become the first genome database with comprehensively functional annotation for bamboo (Zhao et al., 2014). It is also an analytical platform composed of comparative genomic analysis, protein-protein interaction networks, pathway analysis and visualization of genomic data. However, it has only 12 RNA-seq data sets in different tissues of moso bamboo, which falls far short of existing RNA-seq data sets and does not meet the needs of researchers. Moreover, there are no analyses of co-expression networks, functional modules, *cis*-elements and gene set enrichment in BambooGDB. ATTED-II (Aoki et al., 2016), a co-expression database for plant species, provides a view of multiple co-expression data sets for nine species (*Arabidopsis*, field mustard, soybean, barrel medic, poplar, tomato, grape, rice and maize). Only two of them are members of the grass family (Poaceae), like bamboo. It is exceedingly necessary to present co-expression networks for bamboo.

Recently, large amounts of transcriptome data have become available on bamboo for the establishment of co-expression gene networks associated with plant growth and development. We collected 52 high-quality genome-wide transcriptome data sets on moso bamboo covering six tissues from the NCBI SRA database (He et al., 2013; Peng et al., 2013a; Huang et al., 2016; Wei et al., 2016; Zhao et al., 2016, 2018). In addition, we have newly produced 26 in-house transcriptome data sets across six tissues of different growth stages from the Genome Atlas of Bamboo and Rattan (GABR). To efficiently extract information from large data sets, we applied *in silico* methods to build genome-wide global and conditional co-expression networks, and further, to identify functional modules for annotating and predicting bamboo gene functions. Furthermore, we constructed the BambooNET database^1^ to integrate the high-throughput transcriptome data, co-expression networks, functional modules, etc. BambooNET also included co-expression network analysis, *cis*-element analysis and GSEA tools, which might be an online server for refining annotation of bamboo gene functions.

## Materials and Methods

### Moso Bamboo Samples From ICBR

Twenty-six moso bamboo (*Phyllostachys edulis*) samples of ICBR were collected from six main bamboo-producing areas in China during the spring of 2015, including (1) Yixing, Jiangsu Province (N:31°15′08.41″, E:119°43′42.55″, 212 M); (2) Tianmu Mountain, Zhejiang Province (N:30°19′13.42″, E:119°26′55.21″, 480 M); (3) Xianning, Hubei Province (N:29°81′10.02″, E:114°31′21.12″ 150 M); (4) Taojiang, Hunan Province (N:28°28′39.74″, E:112°11′18.62″, 320 M); (5) Guilin, Guangxi Province (N:28°28′39.74″, E:112°11′18.62″, 216 M) and (6) Chishui, Guizhou Province (N:28°28′15.27″, E:105°59′41.43″, 120 M), which covered rhizome, root, shoot, leaf, sheath, and bud during different development stages. Each mixed sample was collected from the above six areas.

### Data Process and Gene Expression Profiling Analysis

The whole-genome sequence of moso bamboo was accessed from the 2013 public version 1 (Peng et al., 2013a) and corresponded to a genome size of ∼2 Gb and 31,987 protein-coding genes. The reads of 78 RNA-seq samples were aligned to the bamboo genome (version 1.0) using TopHat v2.1.1 software (Trapnell et al., 2009). Calculation of FPKM and the identification of differentially expressed genes were performed using Cuffdiff in Cufflinks v2.2.1 software (Trapnell et al., 2010). GO enrichment analysis was performed using the agriGO website (Du et al., 2010).

To determine the minimum threshold of the gene expression value (FPKM) among 78 bamboo samples, the lowest 5% of all gene FPKM values in each RNA-seq sample and the SD of each experimental group were computed. Then, the mathematical formula “threshold = average(5% value) + 3 ^∗^ SD” (You et al., 2016, 2017) was used to calculate the minimum expression value of each experimental group. The minimum threshold of FPKM was 0.1474.

### Co-expression Network Construction Algorithm and Parameters

The PCC represents the co-expression relationship between two genes among the 78 samples. The closer the relationships between the genes were, the higher the PCC scores. MR, an algorithm for calculating the rank of PCC, takes a geometric average of the PCC rank from gene A to gene B and from gene B to gene A. Specifically, when gene A is the third highest co-expressed gene for gene B, the PCC rank of gene A to gene B is 3. Thus, MR ensures more credible co-expression gene pairs would be left out, so the PCC and MR were used to construct a co-expression network.

Pearson correlation coefficient:

(1) $$r_{xy}=\frac{\sum \left(X-\bar{X})(Y-\bar{Y}\right)}{\left(\sqrt{\sum_{i=1}^{n}{\left(X_{i}-\bar{X}\right)}^{2}}\right)\left(\sqrt{\sum_{i=1}^{n}{\left(Y_{i}-\bar{Y}\right)}^{2}}\right)}$$

MR:

(2) $$\mathrm{MR}(\mathrm{AB})=\sqrt{Rank(A\to B)\times Rank(B\to A)}$$

Here, we retained co-expressed gene pairs with a single direction rank of PCC (*Rank*_AB_ or *Rank*_BA_) less than 3 and MR score less than 30 in a co-expression network (Aoki et al., 2016), and these gene pairs were regarded as having positive co-expression relationships when their PCC values were more than zero and negative co-expression relationships when their values were less than zero.

All samples were used to construct global networks, while ICBR samples were used for conditional networks. Following a similar procedure, 65 data sets without the stress treatment were selected to define tissue-preferentially expressed genes, and 10 data sets associated with dehydration and cold treatment were selected for stress-differentially expressed genes.

### Modules Identification Algorithm and Parameters

The CPM (Adamcsek et al., 2006) was used to find modules with nodes more densely connected to each other than to nodes outside the group in the bamboo co-expression networks. Parameter selection was based on the number of modules, the coverage rate of genes and the overlap rate of community. Hence, we selected a *k* = 6 clique size, which meant each node had co-expression interactions with at least five nodes in a module (Supplementary Figure S5). The functions of modules were predicted by gene set analysis (Yi et al., 2013) through integrating annotations such as GO, gene families (transcription regulators, kinases, and carbohydrate-active enzymes), and KEGG. The TF family and kinase family classifications were collected from iTAK (Yi et al., 2016) and PlantTFDB (Jin et al., 2017). A total of 3,305 TFs and 1,598 kinases were identified. Moreover, non-significant entries were filtered by the Fisher’s exact test and multiple hypothesis testing (FDR ≤ 0.05). In the end, 1,896 modules containing at least 6 genes each were found in bamboo, covering functions such as metabolism, hormones, development, and transcriptional regulation.

### *Cis*-Element Significance Analysis

The *cis*-element significance test is a statistical algorithm based on *Z* score and *P*-value filtering. When scanned in the 3 kb promoter region of bamboo genes, motifs with a *P*-value less than 0.05 were significantly enriched in a regulatory module (Yu et al., 2014; You et al., 2016).

The *Z* score was calculated as

$$Z=\frac{\bar{X}-\mu }{\sigma /\sqrt{n}}$$

where $\bar{X}$ is the sum value of a motif in the promoters of a list of genes, μ is the mean value of the same motif in 1000(n) random lists of genes with same scale, and σ is the SD of the 1000-mean value based on random selection.

### Ortholog Identification in *Arabidopsis*

Bidirectional blast alignments were conducted for the analysis of protein sequences in moso bamboo and *Arabidopsis*. Our criteria for the orthologous search were as follows: the top three hits in each bidirectional blast alignment were selected as the best orthologous pairs; in addition, pairs with an *E*-value less than 1E-25 were regarded as secondary orthologous pairs. Table 3 lists the results of the orthologous search, including for NST1, SND1 and VND7.

### Search and Visualization Platform

The network search function was based on MySQL, Apache and PHP scripts. Cytoscape.js, an open source java script package, can dynamically display the components, construction and variation of the network.

## Results

### Network Construction and Module Identification

We integrated 78 transcriptome data sets for moso bamboo (*Phyllostachys edulis*), which can be divided into two parts according to the data source: 52 public data sets from NCBI and 26 in-house data sets from ICBR (Table 1). The data sets spanned most tissues of bamboo, including leaf, culm (stem), shoot, root, rhizome, bud and panicle as well as stress-treated (dehydration and cold) samples from the public platform. The data sets from ICBR were available for the construction of conditional network, covering different portions from tissue root, shoot, bud, leaf and so forth. Furthermore, each ICBR sample was a mixture from six areas of bamboo production in China. This variety is beneficial for the study of fast growth and development regulation in bamboo.

**Table 1:** Details of RNA-seq sample resources.

Tissue	Sample information	Source	Sample number	Reference
Leaf	Transcriptome for photosystems	SRX1035287	3	BMC Plant Biol. 2016;16(1):34 (PMID:26822690)
Leaf	During dehydration and cold stresses	SRS1759772	10	PLoS One.2016;11(11):e0165953 (PMID: 27829056)
Culm (stem)	Transcriptome of developing culms	SRX329521	1	BMC plant biology.2013;13(1): 119 (PMID: 23964682)
Shoot apex (young, 25 cm long)	Shoot apical meristem region	SRS1683502	4	NCBI (2017)


	Young internode region		4	
	Young node region		4	
	Basal mature internode region		4	
	Mature node region		4	
Shoot	Three moso bamboo cultivars(10-15 cm long)	SRP067720	3	NCBI (2016)
Shoot	A thick-wall moso variant and its native wild-type	SRP075216	2	New Phytologist.2016;214(1):81 (PMID: 27859288)
Leaf	Vegetative tissues	ERS123950	2	Nature Genetics.2013;45(4):456 (PMID: 23435089)


Panicle	Panicles at the early stage and flowering stage		4	
Root	Vegetative tissues		2	
Rhizome	Vegetative tissues		2	
Shoot	20-cm-long shoot		1	
Culm	Moso bamboo	SRX342661	2	NCBI (2015)
Root-1	0.1 cm root on shoot	SRP094812	1	Gigascience.2018;doi: 10.1093/gigascience/giy115 (PMID:30202850)
Root-2	0.5 cm root on shoot		1	
Root-3	2 cm root on shoot		1	
Root-4	10 cm root on shoot		1	
Root-5	New root with lateral roots		1	
Root-6	Root on rhizome		1	
Shoot-A1/2/3	Top/middle/lower portion of 0.2 m shoot		3	
Shoot-B1/2/3	Top/middle/lower portion of 1.5 m shoot		3	
Shoot-C1/2/3	Top/middle/lower portion of 3 m shoot		3	
Shoot-D1/2/3	Top/middle/lower portion of 6.7 m shoot		3	
Leaf-1	Blade		1	
Leaf-2	Leaf sheath		1	
Sheath	Sheath sheet		1	
Bud-1/2/3	Bud on top/middle/lower portion of 3 m shoot		3	
Bud-4	Bud on rhizome		1	
Rhizome	Rhizome		1	


A well-developed integrated strategy was used for network construction (You et al., 2016, 2017). First, the raw RNA-seq reads of bamboo samples were mapped to the *moso bamboo* reference genome by TopHat, and then the FPKM of genes were calculated by Cufflinks. Since the read mapping ratios of 7 RNA-seq samples (culm tissue) were too low (<25%), we ultimately retained 78 RNA-seq samples for global co-expression network construction (details of mapping results shown in Supplementary Table S1). Through the FPKM value distribution boxplot of all samples (Supplementary Figure S1), the minimum threshold FPKM value of 0.1474 among 78 bamboo samples was chosen as a cut-off value to identify whether the gene was expressed according to all genes’ FPKM values in each sample. The PCC was adopted as the correlation coefficient between two genes and to measure the co-expression relationship. The lowest 5% PCC (-0.4) and highest 5% PCC values (0.6) were considered thresholds for negative and positive correlation, respectively, in the PCC distribution diagram of all gene pairs (Supplementary Figure S2A). The MR method (Aoki et al., 2016) was widely used in many species such as the model plant *Arabidopsis.* Strict parameters were set to get optimal co-expressed gene pairs in this way. It mainly classified co-expressed gene pairs into three levels: MR top3, MR ≤ 5 and 5 < MR ≤ 30. As a result, the PCC cut-off of 0.6 and the MR top3 + MR cut-off of ≤30 were better for the construction of co-expression network. Finally, there were 302,383 and 185,044 pairs with 31,681 nodes in the positive co-expression network (PCC value > 0) and negative co-expression network (PCC value < 0), respectively. The network was inferred to be scale-free from the distribution of nodes and their linked edges numbers (Supplementary Figure S2B).

All transcriptome data sets were used to construct the global networks in this study, while the 26 data sets from ICBR were used for conditional networks as a parallel analysis with the same method (MR) and procedure as global networks. In addition, 65 data sets without stress treatment were selected to define tissue-preferentially expressed genes, and 10 data sets associated with dehydration and cold treatment were selected for stress-differentially expressed genes. We overlaid the gene expression results onto the co-expression network with multiple dimensions (development and stress).

Furthermore, co-expression networks allow modularized analysis of biological processes to discover regulatory genes or modules of vital traits. The CPM (Adamcsek et al., 2006; Li et al., 2014) together with function enrichment tools was applied to classify possible function modules. As a result, 1,896 functional modules containing at least 6 genes each were identified in bamboo, covering functions such as metabolism, hormones, development, and transcriptional regulation.

### Gene Network Analysis of Photosynthesis-Related Genes

**FIGURE 1:**
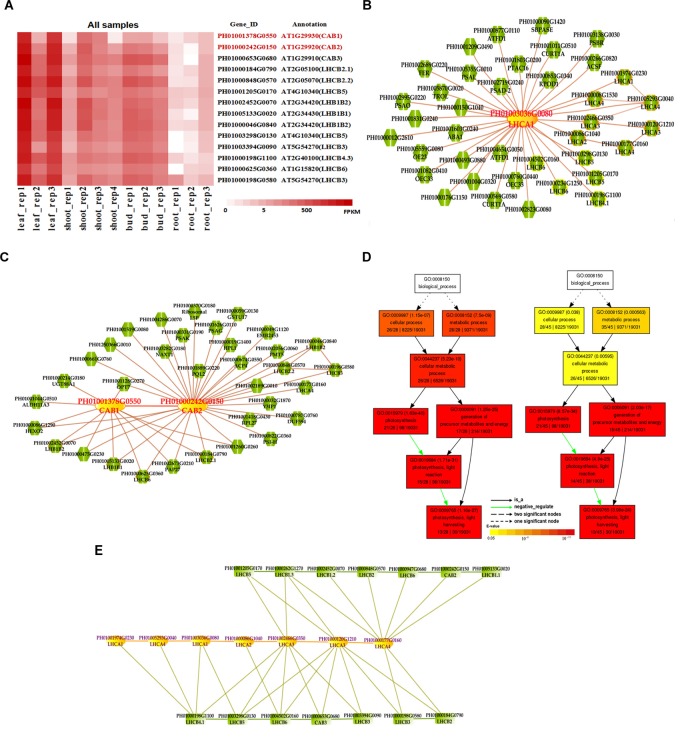
The global co-expression network analysis of bamboo light-harvesting complex genes of photosystem I and photosystem II. **(A)** Heatmap of *LHCB* genes expressed in leaf, shoot, bud, and root samples. **(B)** A global co-expression network of the gene *LHCA1* (PH01003036G0080) in bamboo. The other *LHCA* genes are highlighted with orange borders, while nodes with yellow borders are *LHCB* genes. **(C)** A global co-expression network of *CAB1* (PH01001378G0550, *LHCB1.3*) and *CAB2* (PH01000242G0150, *LHCB1.1*). The other *LHCB* genes are also highlighted with orange borders. **(D)** GO enrichment analysis (left) of all genes from the global co-expression network for *LHCA1* (PH01003036G0080) and GO enrichment analysis (right) of all genes from the global co-expression network for *CAB1* (PH01001378G0550) and *CAB2* (PH01000242G0150) by agriGO. **(E)** The co-expression network between genes *LHCA* and *LHCB* in bamboo.

Photosynthesis provides energy for the fast growth and development of bamboo. It may possess a unique carbon assimilation mechanism, and it would be interesting to study the light-harvesting process in bamboo (Jiang et al., 2012). Additionally, an efficient light-harvesting step is critical for the success of photosynthesis (Cheng and Fleming, 2009; Zhao et al., 2016). We selected three light-harvesting complex (*LHC*) genes of photosystem I and photosystem II in bamboo (Table 2), including PH01003036G0080 (orthologous gene of *LHCA1*), PH01001378G0550 (orthologous gene of *CAB1* or *LHCB1.3*) and PH01000242G0150 (orthologous gene of *CAB2* or *LHCB1.1*), and searched their global co-expression networks with a tissue-preferential gene expression view in bamboo. Based on the *LHCB*-related gene expression views among different tissues, three samples for each tissue were used to detect different expression levels, which were quantified by FPKM value (Figure 1A). These genes preferentially expressed in leaf compared to other tissues, while they were almost not expressed in root. To validate the robustness and credibility of the networks and further study the possible regulatory mechanisms of *LHCA1* and their co-expressed genes in bamboo, we selected the *LHCA1* gene (PH01003036G0080) as an example to visualize the global co-expression network (Figure 1B). Through GO enrichment analysis of all genes from this network by using agriGO (Du et al., 2010; Tian et al., 2017) (Figure 1D), the results showed that these co-expressed genes were strongly associated with the GO terms of photosynthesis and light harvesting, light reaction, and generation of precursor metabolites and energy, which matched the previous findings that the primary function of LHC protein was the absorption of light through chlorophyll excitation and transfer of absorbed energy to photochemical reaction centers (Dolganov et al., 1995; Li et al., 2000; Montané and Kloppstech, 2000; Zhao et al., 2016). A similar result was also obtained in *CAB1, CAB2* and their co-expressed genes following the above process (Figure 1C). Zhao et al. (2016) found that more copies of *LHC* genes indicated more energy may be required in the fast-growth stage of moso bamboo. *LHCA* and *LHCB* coexist with some other *LHC* genes in these global co-expression networks (Figure 1B). From the perspective of only *LHCA* and *LHCB* genes’ co-expression (Figure 1E), *LHCA* and *LHCB* are intimately linked with each other.

**Table 2:** The genes of light-harvesting complex genes of photosystems I and II in bamboo.

Gene ID	Orthologous in *Arabidopsis*	*E*-value		Annotation
PH01003036G0080	AT3G54890	1.1E-99	LHCA1	Light-harvesting complex I chlorophyll a/b binding protein 1
PH01001974G0230	AT3G54890	1E-26	LHCA1	Light-harvesting complex I chlorophyll a/b binding protein 1
PH01000086G1040	AT3G61470	1E-117	LHCA2	Light-harvesting complex I chlorophyll a/b binding protein 2
PH01000120G1210	AT1G61520	7E-109	LHCA3	Light-harvesting complex I chlorophyll a/b binding protein 3
PH01002466G0350	AT1G61520	1E-115	LHCA3	Light-harvesting complex I chlorophyll a/b binding protein 3
PH01000008G1530	AT3G47470	8E-108	LHCA4	Light-harvesting complex I chlorophyll a/b binding protein 4
PH01000177G0160	AT3G47470	1E-106	LHCA4	Light-harvesting complex I chlorophyll a/b binding protein 4
PH01005293G0040	AT3G47470	1E-107	LHCA4	Light-harvesting complex I chlorophyll a/b binding protein 4
PH01000173G0670	AT1G45474	7.6E-83	LHCA5	Light-harvesting complex I chlorophyll a/b binding protein 5
PH01001378G0550	AT1G29930	2E-132	LHCB1.3	AB140, **CAB1**, CAB140, chlorophyll a/b binding protein1, LHCB1.3, light-harvesting chlorophyll a/b protein1.3
PH01000242G0150	AT1G29920	9E-134	LHCB1.1	AB165, **CAB2**, chlorophyll a/b binding protein2, LHCB1.1, light-harvesting chlorophyll a/b protein1.1
PH01000653G0680	AT1G29910	8E-135	LHCB1.2	AB180, **CAB3**, chlorophyll a/b binding protein3, LHCB1.2, light harvesting chlorophyll a/b binding protein1.2
PH01000625G0360	AT1G15820	3.00E-111	LHCB6	CP24, LHCB6, light harvesting complex photosystem II subunit 6
PH01002452G0070	AT2G34420	1.00E-128	LHB1	Light-harvesting complex II chlorophyll a/b binding protein 1
PH01000046G0840	AT2G34420	2.40E-99	LHB1B2	Light-harvesting complex II chlorophyll a/b binding protein 1
PH01005133G0020	AT2G34430	7.00E-131	LHB1B1	Light-harvesting complex II chlorophyll a/b binding protein 1
PH01000848G0570	AT2G05070	3.00E-120	LHCB2.2	Light-harvesting complex II chlorophyll a/b binding protein 2
PH01000184G0790	AT2G05100	7.00E-120	LHCB2.1	Light-harvesting complex II chlorophyll a/b binding protein 2
PH01000848G0570	AT3G27690	3.00E-120	LHCB2.3	Light-harvesting complex II chlorophyll a/b binding protein 2
PH01000198G0580	AT5G54270	4.00E-135	LHCB3	Light-harvesting complex II chlorophyll a/b binding protein 3
PH01003394G0090	AT5G54270	4.00E-134	LHCB3	Light-harvesting complex II chlorophyll a/b binding protein 3
PH01000198G1100	AT2G40100	2.00E-102	LHCB4	Light-harvesting complex II chlorophyll a/b binding protein 4
PH01001205G0170	AT4G10340	2.00E-112	LHCB5	Light-harvesting complex II chlorophyll a/b binding protein 5
PH01003298G0130	AT4G10340	9.00E-108	LHCB5	Light-harvesting complex II chlorophyll a/b binding protein 5


**FIGURE 2:**
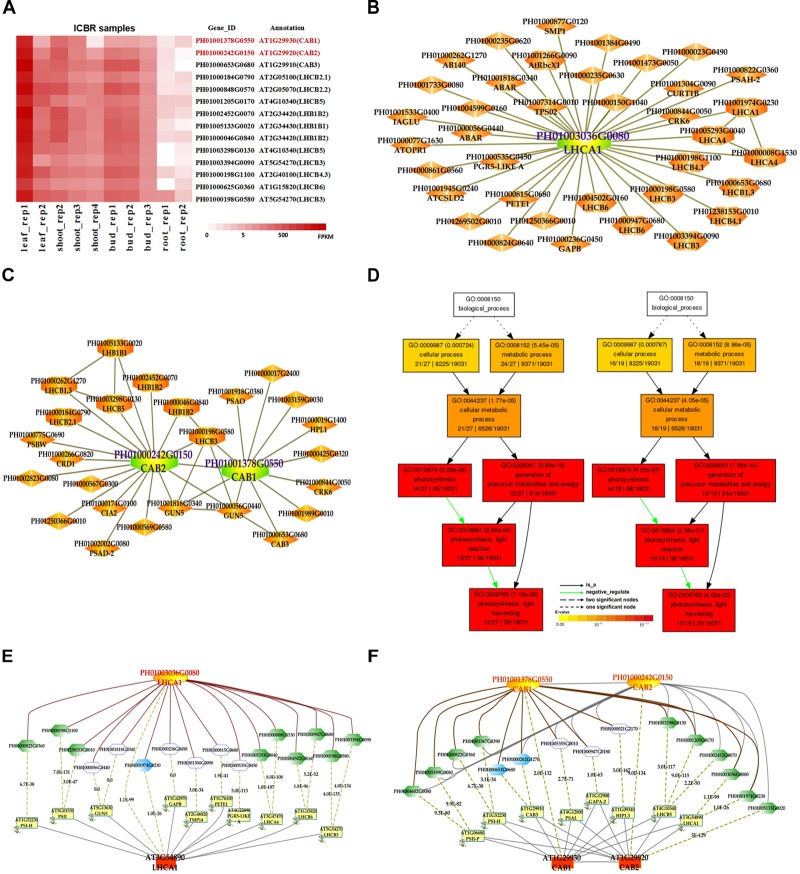
The conditional co-expression network analysis of bamboo light-harvesting complex genes of photosystem I and photosystem II. **(A)** Heatmap of *LHCB* genes expressed in conditional leaf, shoot, bud, and root samples. **(B)** A conditional co-expression network of the gene *LHCA1* (PH01003036G0080) in bamboo. The other *LHCA* genes are highlighted with orange borders, while nodes with yellow borders are *LHCB* genes. **(C)** A conditional co-expression network of *CAB1* (PH01001378G0550, *LHCB1.3*) and *CAB2* (PH01000242G0150, *LHCB1.1*). The other *LHCB* genes are also highlighted with orange borders. **(D)** GO enrichment analysis (left) of all genes from the conditional co-expression network for *LHCA1* (PH01003036G0080) and GO enrichment analysis (right) of all genes from the conditional co-expression network for *CAB1* (PH01001378G0550) and *CAB2* (PH01000242G0150) by agriGO. **(E)** Comparison of conditional co-expression networks between *LHCA1* in bamboo and AT3G54890 (*LHCA1*) in *Arabidopsis*. Dotted lines link orthologous pairs between the two species, and the numbers in the middle of the dotted lines are the *E*-values of the BLAST results. **(F)** Comparison of conditional co-expression networks between *CAB1* and *CAB2* in bamboo and AT1G29930 (*CAB1*) and AT1G29920 (*CAB2*) in *Arabidopsis*. Dotted lines link orthologous pairs between the two species, and the numbers in the middle of the dotted lines are the *E*-values of the BLAST results.

In addition, we also searched the co-expression network of photosynthesis-related genes in the conditional co-expression network. We performed the same procedure for the global network analysis as in the conditional network of *LHC* genes. The gene expression views in the conditional network (Figure 2A) showed a similar tendency to those in the global network. Meanwhile, we conducted GO enrichment analysis of all genes from the conditional co-expression network for *LHCA1* (PH01003036G0080), *CAB1* (PH01001378G0550) and *CAB2* (PH01000242G0150) by agriGO (Du et al., 2010; Tian et al., 2017). The GO terms were associated with photosynthesis, light reaction and light harvesting (Figure 2D). In addition to overlaps, the conditional co-expression network had some specific genes that were different from the global network (Figures 2B,C).

Comparative genomics might help to construct and identify functional modules in bamboo. We made a comparison between the top 300 PCC co-expressed genes in bamboo and in *Arabidopsis* (collected from ATTED-II and AraNet) (Figures 2E,F). The co-expression networks of PH01003036G0080 and AT3G54890 (*LHCA1*) showed high similarity, suggesting the reliability of our bamboo co-expression network.

### Network Analysis of Genes Related to Brassinosteroid Biosynthetic and Signal Transduction Pathways

**FIGURE 3:**
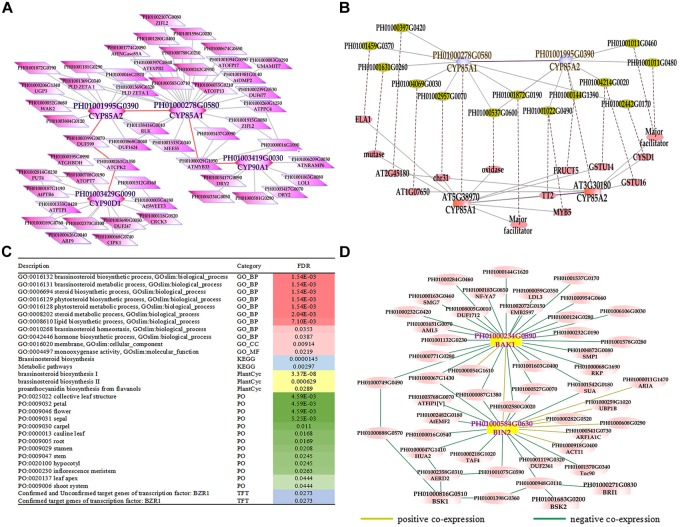
The global co-expression network analysis of bamboo brassinosteroid (BR) biosynthesis genes and signal transduction genes. **(A)** A global co-expression network of BR biosynthesis genes *CYP85A1* (PH01000278G0580), *CYP85A2* (PH01001995G0390), *CYP90A1* (PH01003419G0030) and *CYP90D1* (PH01003429G0090) in bamboo. **(B)** Comparison of global co-expression networks between BRs biosynthesis genes *CYP85A1* and *CYP85A2* in bamboo and AT5G38970 (*CYP85A1*) and AT3G30180 (*CYP85A2*) in *Arabidopsis*. Dotted lines link orthologous pairs between the two species. **(C)** GSEA analysis of all genes from Figure 3A. The results shown in this table list the description, category and FDR value. The red bar on the right represents the FDR value of the enriched GO terms. The darker the red color becomes, the lower the FDR value is. Other colored bars, including blue, yellow, green, and purple bars, represent KEGG pathways, Plantcyc, PO terms and TFT, respectively. **(D)** A global co-expression network of BR signal transduction genes *BAK1* (PH01000234G0890) and *BIN2* (PH01000584G0630) in bamboo. A yellow line between two nodes indicates a positive co-expression relationship, and a green line between two nodes indicates a negative co-expression relationship.

Phytohormones are indispensable in plant development and various environment adaption (Lacombe and Achard, 2016). BRs are a group of plant steroidal hormones that play vital roles in almost all aspects of plant growth and development (Du et al., 2017). Several key enzymes in BR biosynthesis pathways have been found in *Arabidopsis*, such as DET2/DWF6, CYP90B1/DWF4, CYP90A1/CPD/DWF3, CYP90C1/ROT3, CYP90D1, and CYP85A2/BR6OX2 (Fujioka et al., 1997; Choe et al., 1998; Yukihisa et al., 2003; Kim et al., 2005; Ohnishi et al., 2012). First, we searched the global network for gene PH01003419G0030 (orthologous gene of *CYP90A1/CPD/DWF3*), PH01000278G0580 (orthologous gene of *CYP85A1/BR6OX1*), PH01001995G0390 (orthologous gene of *CYP85A2/BR6OX2*), and PH01003429G0090 (orthologous gene of *CYP90D1*) (Figure 3A). Second, we conducted GSEA analysis of GO, gene family, PlantCyc and KEGG categories for all genes from this global network by using PlantGSEA (Yi et al., 2013) (Figure 3C). The GO terms of BR biosynthetic process, BR metabolic process and steroid biosynthetic process were significantly enriched, suggesting that this network corresponds to the BR biosynthetic pathway and the genes from this network may be involved in BR biosynthesis in bamboo. Third, we chose the genes *CYP85A1/BR6OX1* and *CYP85A2/BR6OX2* in bamboo and their top 300 co-expressed genes and compared them with their orthologous genes and their top 300 from ATTED-II in *Arabidopsis* (Figure 3B). There were many orthologous gene pairs between them, which could indicate these co-expressed genes were conserved and increased the credibility of predicting BR biosynthetic functional modules in bamboo. We also searched the global network for PH01000234G0890 (orthologous gene of *BAK1*, also known as BRI1-associated receptor kinase), PH01000584G0630 (orthologous gene of *BIN2*, also known as BR-insensitive 2) and their co-expressed genes (Figure 3D). With the GO enrichment analysis by agriGO on all the genes from this network, some GO terms were enriched such as the BR-mediated signaling pathway, steroid hormon mediated signaling pathway and responses to steroid hormone stimuli (Supplementary Figure S4).

### Co-expression Network Analysis of Secondary Cell Wall Biosynthesis

**FIGURE 4:**
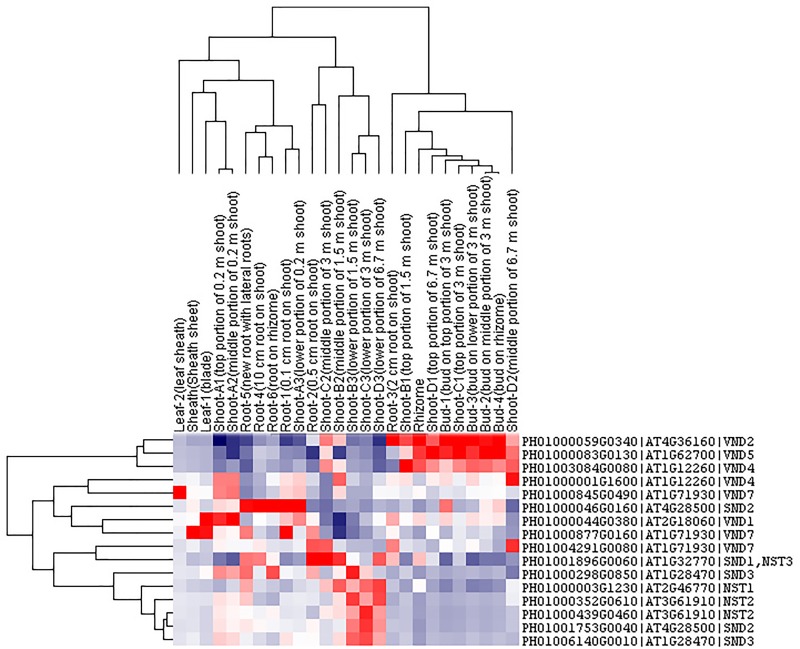
Cluster analysis of *SND, NST* and *VND* genes from ICBR samples of bamboo. The gene names are indicated on the right, while the tissue types are shown above each column. High, average and low levels of expression in a specific tissue are indicated by red, white, and blue, respectively.

**FIGURE 5:**
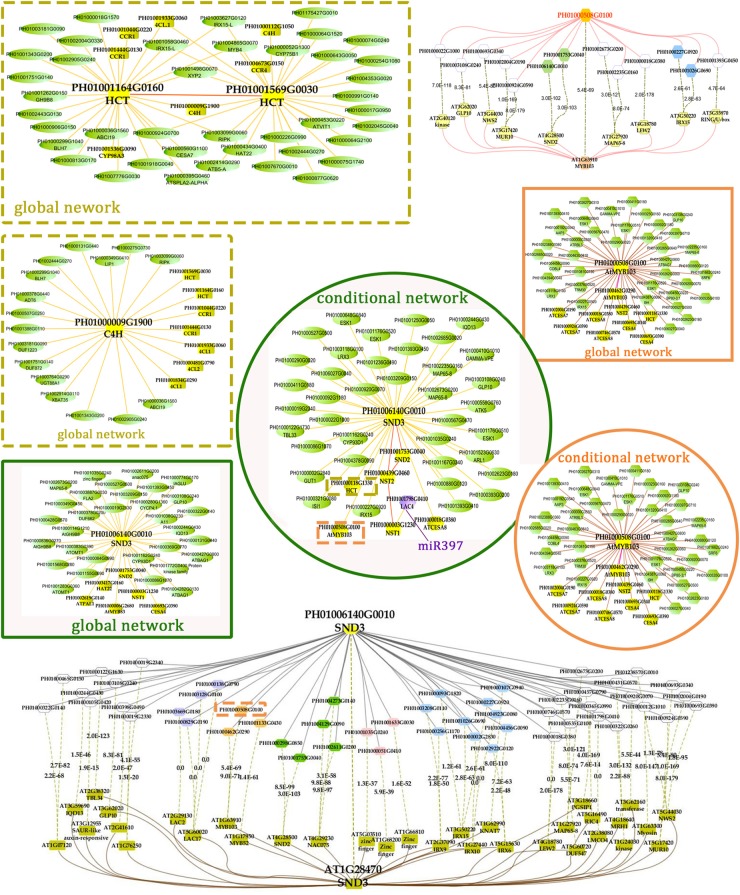
A co-regulatory network for *NSTs/SNDs*-related genes in bamboo. PH01006140G0010 (*SND3*) and PH01000508G0100 (orthologous to *AtMYB103*) were used to present regulatory networks with all samples (global network) and partial samples (conditional network). The rounded boxes represent conditional networks, while the rectangular boxes represent global networks. The networks for the genes PH01006140G0010 (*SND3*) and PH01000508G0100 (orthologous to *AtMYB103*) are in the boxes with green solid borders and orange solid borders, respectively. The networks for *HCT* (PH01001164G0160 and PH01001569G0030) and *C4H* (PH01000009G1900) are in the boxes with yellow-dotted borders. The comparison views of conditional co-expression networks are also shown. The gray edges link to *SND3* in bamboo, and brown edges link to AT1G28470 (*SND3*) in *Arabidopsis*. The red edges link the *AtMYB103* (AT1G63910) gene and orthologs of *AtMYB103* in bamboo (PH01000508G0100). Dotted lines link orthologous pairs between the two species, and the numbers in the middle of the dotted lines are the *E*-values of the BLAST results. The *NAC, MYB*, zinc finger, *IRX* and *LAC* genes are highlighted with green, orange, pink, blue, and purple nodes, respectively. In the conditional co-expression network for PH01006140G0010 (*SND3*), the *LAC4* gene PH01001798G0410 is highlighted in purple, which is a miR397 target gene.

Transcription factors in the NAC family, including VNDs, SNDs, and NSTs, acting as master switches for SCW thickening, play important roles in the SCW formation process, including the deposition of hemicellulose, cellulose and lignin (Table 3). MYB46 directly binds to the promoters and activates the transcription of genes involved in lignin and xylan biosynthesis, functioning as a central and direct regulator of the genes involved in the biosynthesis of all three major secondary wall components in *Arabidopsis* (Kim et al., 2013, 2014a). Thus, we selected some key NAC and MYB TFs to study their functions in regulating SCW formation and strong lignified culms in bamboo (Table 4). The gene expression profiling of SCW-related NAC family genes was statistically analyzed with the *Z*-score test in ICBR samples. The hierarchical cluster results of these genes demonstrated that *NST/SND* genes were highly expressed in the bamboo shoot compared to other tissues (Figure 4). We searched the constructed global and conditional networks with a gene expression view for these clustered *NAC* genes. The networks might indicate the possible regulatory mechanism of the SCW thickening process during bamboo development (Figure 5).

**Table 3:** Information of NAC family in bamboo.

Gene ID	Subfamily	Orthologous in *Arabidopsis*	*E*-value
PH01000439G0460	NST2, ANAC066	AT3G61910	3.4E-80
PH01001896G0060	SND1, NST3	AT1G32770	2.9E-80
PH01000003G1230	NST1, EMB2301	AT2G46770	8.3E-91
PH01000352G0610	NST2, ANAC066	AT3G61910	1.7E-79
PH01000298G0850	SND3, ANAC010	AT1G28470	4.90E-89
PH01001753G0040	SND2, ANAC073	AT4G28500	3E-103
PH01006140G0010	SND3, ANAC010	AT1G28470	9.2E-88
PH01000046G0160	SND2, ANAC073	AT4G28500	1E-91
PH01000059G0340	VND2	AT4G36160	4.3E-87
PH01000001G1600	VND4	AT1G12260	2E-98
PH01000044G0380	VND1	AT2G18060	1.5E-74
PH01000877G0160	VND7	AT1G71930	9.2E-48
PH01004291G0080	VND7	AT1G71930	9.80E-47
PH01003084G0080	VND4	AT1G12260	1.50E-77
PH01000845G0490	VND7	AT1G71930	6E-79
PH01000083G0130	VND5	AT1G62700	3.40E-63


**Table 4:** Information of MYB family in bamboo.

Gene ID	Subfamily	Orthologous in * Arabidopsis*	*E*-value
PH01002276G0160	ATMYB80	AT5G56110	6.8E-54
PH01000041G2150	ATMYB80	AT5G56110	2E-53
PH01000198G1320	ATMYB80	AT5G56110	4.4E-53
PH01000060G0800	MYB85	AT4G22680	2.30E-73
PH01000427G0040	MYB42	AT4G12350	6E-67
PH01001430G0250	MYB85	AT4G22680	1.7E-66
PH01003093G0130	MYB85	AT4G22680	5.1E-70
PH01128678G0010	MYB69	AT4G33450	3.5E-49
PH01002104G0150	MYB52	AT1G17950	4E-53
PH01002184G0220	MYB63	AT1G79180	2.4E-42
PH01000030G0050	MYB63	AT1G79180	2.1E-62
PH01000386G0660	MYB58	AT1G16490	3.2E-54
PH01001133G0430	MYB54	AT1G73410	6E-54
PH01000006G2680	MYB46	AT5G12870	1.5E-54
PH01000008G3080	MYB20	AT1G66230	5.5E-73
PH01005828G0060	MYB43	AT5G16600	4.10E-76
PH01000847G0490	MYB43	AT5G16600	7.7E-69
PH01000569G0800	MYB20	AT1G66230	4.2E-68
PH01001342G0270	MYB20	AT1G66230	3.3E-70
PH01000462G0290	AtMYB103	AT1G63910	9E-71
PH01000508G0100	AtMYB103	AT1G63910	5.4E-69


In the conditional network, some *NST/SND* genes, such as PH01006140G0010 (*SND3*), PH01001753G0040 (*SND2*), PH01000439G0460 (*NST2*) and PH01000003G1230 (*NST1*), were co-expressed together with *LAC4*, one of the key SCW metabolism-related genes. Xu et al. (2014) has discovered that the putative targets of miRNA-397 are several family members of laccase precursors in bamboo, including the *LAC4* gene PH01001798G0410. Additionally, *SND3* was co-expressed with the IRREGULAR XYLEM gene *IRX15* (PH01000227G0920), *AtMYB103* in bamboo (PH01000508G0100), *AtCESA8* in bamboo (PH01000018G0380) and the phenylpropanoid biosynthesis pathway gene *HCT* (PH01000118G1330).

In the global network, *SND3* was co-expressed with *SND2* and *NST1, CESA4, HAT22, PAL1* and an ortholog of *AtMYB83* in bamboo (PH01000006G2680). In *Arabidopsis*, both MYB46 and MYB83 act in the regulation of secondary wall biosynthesis by binding to the promoters of the xylan and lignin biosynthetic genes (Mccarthy et al., 2009; Kim et al., 2014b).

With regard to the global network for PH01000508G0100 (orthologous gene of *AtMYB103*), the co-expressed genes were mainly *HCT, ATCESA7, ATCESA8, CESA4*, and some *NAC* genes *SND2, SND3*. There were a few specific co-expressed genes, the *NAC* gene *NST2* and another ortholog of *AtMYB103* in bamboo (PH01000462G0290). Meanwhile, we supplied a visualization of global networks for some genes related to the phenylpropanoid biosynthesis pathway, such as PH01001164G0160 (orthologous gene of *HCT*), PH01001569G0030 (orthologous gene of *HCT*) and PH01000009G1900 (orthologous gene of *C4H*) (Figure 5). These genes were also co-expressed with some genes related to phenylpropanoid biosynthesis pathways, including *CCR1, CCR4, 4CL1, 4CL2*, and *CYP98A*, whose promoter regions share a *cis*-acting motif called ‘AC element’ that is recognized by MYB58 and MYB63 in *Arabidopsis* (Zhou et al., 2009). Through motif analysis of 3 kb of these bamboo genes’ promoter regions, the ‘AC element’ was found to be significantly enriched.

To increase the reliability of networks in bamboo, we further made a comparison between the top 300 PCC co-expressed genes of *SND3* and *MYB103* in Arabidopsis (from ATTED-II) and those in bamboo. Plenty of orthologous gene pairs in *SND3* network comparison could be grouped into several sections, such as *LAC* genes, *MYB* genes, zinc finger genes, *IRX* family genes and other *NAC* genes. Generally, our co-expression network analysis, together with the *cis*-element and GO enrichment analyses, efficiently identified components and recapitulated a regulatory module of the SCW biosynthetic process.

### A Combination of Several Functional Regulatory Modules Related to Bamboo Development

**FIGURE 6:**
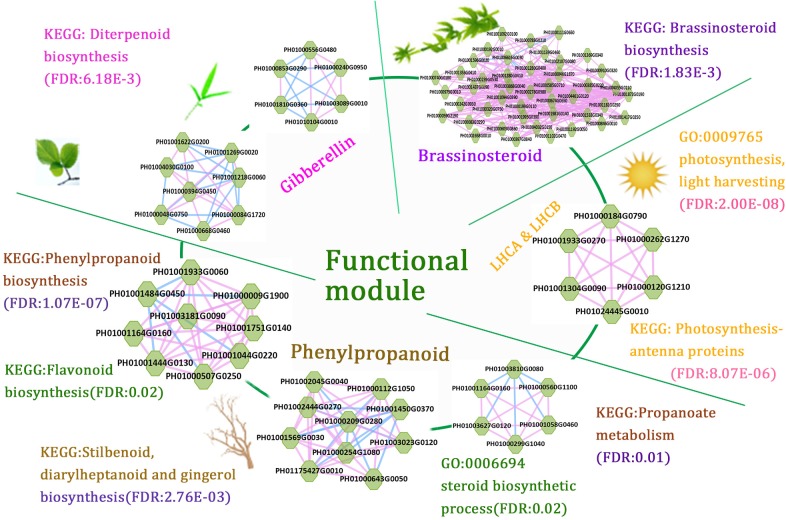
A combination of functional regulatory modules related to bamboo development through module prediction. The functions of the modules were predicted through integrating annotations such as GO, gene families (transcription regulators, kinases, and carbohydrate-active enzymes), and KEGG, and non-significant entries were filtered by the Fisher test and multiple hypothesis testing (FDR ≤ 0.05). The different modules with similar functions are in the same section.

The function modules contained nodes that were more densely connected to each other than to nodes outside the group in bamboo co-expression networks. We identified an important functional module related to photosynthesis by co-expression network analysis, and the function of this module was predicted to associate with photosynthesis and light harvesting (FDR: 2.00E-8) by GSEA (Figure 6). We also identified functional modules related to BR biosynthetic pathways (FDR: 1.83E-3) and diterpenoid biosynthetic pathways (FDR: 6.18E-3) based on a similar approach (Figure 6). In addition, three regulatory modules were found to possibly participate in phenylpropanoid biosynthetic pathways. For example, one functional module was significantly related to phenylpropanoid biosynthesis (FDR: 1.07E-7) and flavonoid biosynthesis (FDR: 0.02), including PH01000009G1900 (orthologous gene of *C4H*), PH01001044G0220 (orthologous gene of *CCR1*), and PH01001444G0130 (orthologous gene of *CCR1*).

We further combined several regulatory gene modules that were identified and conducted module analysis of functions related to fast growth of bamboo culms (Figure 6). All these modules were related to bamboo growth and development, such as photosynthesis, BR biosynthesis, and phenylpropanoid biosynthesis. Among the different modules related to phenylpropanoid biosynthesis, the connected node (PH01001164G0160, orthologous gene of *HCT*) could play a vital role in the regulatory pathway based on its co-expression network analysis. The connections between functional modules could represent crosslinks between different modules related to the similar function or different pathways. Thus, modules with nodes connected to other modules were selected and displayed in the database to help to further study their key functions. Accordingly, the combination of these functional modules displayed a series of possible key genes from hormone signals to culm development, mimicking the dynamic regulatory process in bamboo and highlighting the connections between these nodes in regulatory modules during growth stages.

### Online Co-expression Network Database for Moso Bamboo

Here, we developed the BambooNET database, a platform with co-expression network analysis, *cis*-element analysis and GSEA tools and provided an online server for gene functional module analyses in multi-dimensional co-expression networks for moso bamboo (*Phyllostachys edulis*), which will help to refine annotation of bamboo gene functions. In this database, different categories of the co-expression network can be selected to visualize using the Cytoscape web tool, including the global network and the conditional network, which includes a search function for either a single gene or a list of genes. Notably, there are three main analysis options in the co-expression network platform: positive relationship, negative relationship and predicted protein-protein interaction relationship. In the tissue-preferential analysis, there are eight tissues, namely, the shoot, root, culm (stem), leaf, panicle, bud, rhizome and sheath. In addition, the gene expression changes of a certain sub-network among different tissues can be clearly observed. The stress-differential analysis displays not only the differences in gene expression between 2 and 8 h under dehydration and cold stress but also the fold changes of gene expression after each stress treatment. In the view of the network, the nodes in red or blue represent up- or down-regulated genes in leaves after a stress treatment, respectively. Moreover, some tools in this platform are available for the gene lists from the selected specific network to analyze, annotate and identify some functional modules, besides gene set analysis (Yi et al., 2013) and UCSC Genome Browser (Speir et al., 2016), such as BLAST search, keyword search and module enrichment analysis, comprising co-expression network and miRNA-target network. The website can be accessed at http://bioinformatics.cau.edu.cn/bamboo.

## Discussion

In this study, we constructed a genome-level co-expression network containing more than 90% of predicted genes and 487,427 positive and negative edges with existing transcriptome data on bamboo. The samples cover most of the development stages of bamboo growth and development, such as the root, leaf, culm (stem), and shoot. In addition, a network-based platform, covering global, conditional and predicted protein-protein interaction networks, has been built successfully to refine the annotation of bamboo genes or modules with functions related to bamboo growth and development. Through the data mining system, networks of various aspects have combined with several functional analysis tools, including ortholog annotation, gene family classification, *cis*-element analysis and GO analysis, to evaluate the reliability of the predictions.

Although the whole-genome sequence of moso bamboo has been released, the genome annotation is still far from complete. For these sparsely annotated genes, compared with the study of single-gene identifications, modules are a valuable resource for predicting gene function. Combined with genes from co-expression networks, it would be interesting to identify modules associated with the biological process of bamboo growth and development. The potential functional modules related to phytohormones can display the module functions for BR biosynthetic pathways (FDR: 1.83E-3) and diterpenoid biosynthetic pathways (FDR: 6.18E-3) by GSEA analysis of KEGG pathways (Figure 6). These tightly linked genes within the one module may have related key biological functions in the process of fast growth in bamboo and can be used for genetic improvement and molecular regulatory mechanisms of moso bamboo. There is great potential for producing a large number of mutant traits of target genes related to bamboo growth and development using the clustered regularly interspaced short palindromic repeats (CRISPR)-associated protein 9 (CRISPR/Cas9)-based genome-editing systems. Natural variants of these genes in different bamboo species may also be favorable for genetic improvement of traits in crops. Compared with the model plant *Arabidopsis*, the similar functions of orthologous genes have validated the credibility of the above network analysis and module implications based on the network comparison between *Arabidopsis* and bamboo (Figure 3B).

The co-expression networks with different samples are usually different. An ideal method should be able to incorporate global networks and conditional networks for different samples. Compared with all samples, there is much less diversity and far more comparability between ICBR samples (Supplementary Figure S3). Moreover, all ICBR samples should be classified as vegetative tissues, which may have parts specifically related to fast growth and the development of shoot and culm. For the co-expression networks for *LHC* genes (Figures 2B,C), the genes between global and conditional networks have their differences and overlaps. Therefore, the conditional co-expression network together with the global network can be complementary and then imitate the potential *LHC* genes’ regulatory mechanism of fast-growth stage in bamboo. Specifically, we also investigated whether network modules are associated with specific tissue types and are enriched for specific biological process analysis by agriGO based on cluster analysis of *SND, NST* and *VND* genes between conditional samples (from ICBR) and global samples (all source) of bamboo. These genes are preferentially expressed within shoot tissues relative to all other tissue types in conditional samples (Figure 4), which would be essential in the growth of bamboo, especially the tissue shoot. For example, one regulatory module with the gene *SND3* for SCW thickening was identified based on the conditional co-expression networks, which might indicate that these genes can fulfill their function in shoot development stages. Meanwhile, the global networks provided additional genes for further exploration of shoot tissue development.

Although BambooGDB (Zhao et al., 2014) has been integrated high-throughput sequencing data and provided researchers worldwide with a central genomic resource and an extensible analysis platform for bamboo genome, it is still necessary to build an online database for refining gene annotation and discovering novel gene functions. Through Cytoscape, our online bamboo co-expression database displays the multi-dimensional network structure and module enrichment for clear visualization and convenient analysis. Based on the co-expression network, the strategy for functional module prediction and refined gene function annotation is general and effective, so more regulatory modules could be identified by the same strategy based on a detailed biological focus or event, such as fast growth. We successfully identified 1,896 functional modules through the CPM method (Adamcsek et al., 2006), which can be searched and studied through the module enrichment in the database. These findings make it more convenient to understand the molecular regulatory mechanisms of bamboo’s vital developmental traits, extremely its fast growth, which can help to dissect the molecular biological processes of bamboo. In addition, the unannotated genes establish connections to their co-expressed genes and can be refined by functional module enrichment analysis to further study unknown functions with biological processes and discern gene transcriptional regulatory mechanisms *in vivo* with the help of gene expression view in different tissues. With our multi-dimensional co-expression network, more than 90% of unannotated bamboo genes might be predicted potential functions.

However, the results might be unsatisfactory owing to the lack of complete data sets on all kinds of tissues in bamboo development stages. We believe that the detection of function modules will become much more efficient with more comprehensive transcriptome data sets of moso bamboo. Furthermore, our online bamboo co-expression database will be improved to facilitate data visualization. We will further incorporate faster and more efficient tools, such as JBrowse (Buels et al., 2016), which are very convenient for genomic track data visualization. Finally, we expect to functionally characterize modules and to investigate how to alter modules to drive developmental changes across all developmental stages and how genes in these modules act in biological pathways.

## Conclusion

Here, multi-dimensional bamboo samples and comparable computing measurements have been used to build a co-expression network to refine the annotation of bamboo genes or functional modules with important agronomic traits, such as growth processes. Meanwhile, module functional enrichment analysis tools, such as gene family classification, *cis*-element analysis and GO analysis, have been used to evaluate the reliability of the predictions. Based on the gene expression analysis and conditional network, the strategy for functional module prediction and refined gene function annotation is general and effective. Thus, more regulatory modules could be identified by the same strategy based on a detailed biological focus or event, such as fast growth. Therefore, this approach will improve our understanding of the molecular regulatory mechanisms underlying vital agronomic traits, such as growth and development. We hope that more transcriptome data will improve the network analysis for functional module identification and reduce biases or mistakes caused by its current limitations, increasing our understanding of bamboo growth and development.

## Author Contributions

ZS, WX, and ZG designed the project. XM, HZ, WX, and HY performed the research. XM, QY, and WX analyzed the data and conducted the bioinformatics analysis. XM, HZ, WX, ZG, and ZS wrote the article.

## Conflict of Interest Statement

The authors declare that the research was conducted in the absence of any commercial or financial relationships that could be construed as a potential conflict of interest.

## Footnotes

1 http://bioinformatics.cau.edu.cn/bamboo

## Abbreviations

BR: brassinosteroid
CPD: CONSTITUTIVE PHOTOMORPHOGENESIS AND DWARFISM
CPM: clique percolation method
DET2: DE-ETIOLATED2
FDR: false discovery rate
FPKM: fragments per kilobase of transcript per million mapped reads
GO: Gene Ontology
GSEA: gene set enrichment analysis
ICBR: International Center for Bamboo and Rattan
KEGG: Kyoto Encyclopedia of Genes and Genomes
MR: mutual rank
PCC: Pearson correlation coefficient
ROT3: CYP90C1/ROTUNDIFOLIA
SCW: secondary cell wall
SD: standard deviation
TF: transcription factor

## References

[B1] Adamcsek B. Palla G. Farkas I. J. Derényi I. Vicsek T. (2006). CFinder: locating cliques and overlapping modules in biological networks. *Bioinformatics* 22 1021–1023. 10.1093/bioinformatics/btl039 16473872

[B2] Aoki K. Ogata Y. Shibata D. (2007). Approaches for extracting practical information from gene co-expression networks in plant biology. *Plant Cell Physiol.* 48 381–390. 10.1093/pcp/pcm013 17251202

[B3] Aoki Y. Okamura Y. Tadaka S. Kinoshita K. Obayashi T. (2016). ATTED-II in 2016: a plant coexpression database towards lineage-specific coexpression. *Plant Cell Physiol.* 57:e5. 10.1093/pcp/pcv165 26546318 PMC4722172

[B4] Bassel G. W. Glaab E. Marquez J. Holdsworth M. J. Bacardit J. (2011). Functional network construction in arabidopsis using rule-based machine learning on large-scale data sets. *Plant Cell* 23 3101–3116. 10.1105/tpc.111.088153 21896882 PMC3203449

[B5] Buels R. Yao E. Diesh C. M. Hayes R. D. Munoz-Torres M. Helt G. (2016). JBrowse: a dynamic web platform for genome visualization and analysis. *Genome Biol.* 17:66. 10.1186/s13059-016-0924-1 27072794 PMC4830012

[B6] Cheng Y. C. Fleming G. R. (2009). Dynamics of light harvesting in photosynthesis. *Annu. Rev. Phys. Chem.* 60 241–262. 10.1146/annurev.physchem.040808.090259 18999996

[B7] Choe S. Dilkes B. P. Fujioka S. Takatsuto S. Sakurai A. Feldmann K. A. (1998). The DWF4 gene of *Arabidopsis* encodes a cytochrome P450 that mediates multiple 22alpha-hydroxylation steps in brassinosteroid biosynthesis. *Plant Cell* 10 231–243. 10.1105/tpc.10.2.231 9490746 PMC143988

[B8] D’Haeseleer P. Liang S. Somogyi R. (2000). Genetic network inference: from co-expression clustering to reverse engineering. *Bioinformatics* 16 707–726. 10.1093/bioinformatics/16.8.707 11099257

[B9] Dolganov N. A. Bhaya D. Grossman A. R. (1995). Cyanobacterial protein with similarity to the chlorophyll a/b binding proteins of higher plants: evolution and regulation. *Proc. Natl. Acad. Sci. U.S.A.* 92 636–640. 10.1073/pnas.92.2.636 7831342 PMC42797

[B10] Du J. Zhao B. Sun X. Sun M. Zhang D. Zhang S. (2017). Identification and characterization of multiple intermediate alleles of the key genes regulating brassinosteroid biosynthesis pathways. *Front. Plant Sci.* 7:1893. 10.3389/fpls.2016.01893 28138331 PMC5238361

[B11] Du Z. Zhou X. Ling Y. Zhang Z. Su Z. (2010). agriGO: a GO analysis toolkit for the agricultural community. *Nucleic Acids Res.* 38 W64–W70. 10.1093/nar/gkq310 20435677 PMC2896167

[B12] Fujioka S. Li J. Choi Y. H. Seto H. Takatsuto S. Noguchi T. (1997). The Arabidopsis deetiolated2 mutant is blocked early in brassinosteroid biosynthesis. *Plant Cell* 9 1951–1962. 10.1105/tpc.9.11.1951 9401120 PMC157049

[B13] He C. Cui K. Zhang J. Duan A. Zeng Y. (2013). Next-generation sequencing-based mRNA and microRNA expression profiling analysis revealed pathways involved in the rapid growth of developing culms in moso bamboo. *BMC Plant Biol.* 13:119. 10.1186/1471-2229-13-119 23964682 PMC3765735

[B14] Huang Z. Zhong X. J. He J. Jin S. H. Guo H. D. Yu X. F. (2016). Genome-wide identification, characterization, and stress-responsive expression profiling of genes encoding lea (late embryogenesis abundant) proteins in moso bamboo (*Phyllostachys edulis*). *PLoS One* 11:e0165953. 10.1371/journal.pone.0165953 27829056 PMC5102402

[B15] Jiang Z. H. Peng Z. H. Gao Z. M. Liu C. Yang C. H. (2012). Characterization of different isoforms of the light-harvesting chlorophyll a/b complexes of photosystem II in bamboo. *Photosynthetica* 50 129–138. 10.1007/s11099-012-0009-7

[B16] Jin J. Tian F. Yang D. C. Meng Y. Q. Kong L. Luo J. (2017). PlantTFDB 4.0: toward a central hub for transcription factors and regulatory interactions in plants. *Nucleic Acids Res.* 45 D1040–D1045. 10.1093/nar/gkw982 27924042 PMC5210657

[B17] Kim G. T. Fujioka S. Kozuka T. Tax F. E. Takatsuto S. Yoshida S. (2005). CYP90C1 and CYP90D1 are involved in different steps in the brassinosteroid biosynthesis pathway in *Arabidopsis thaliana*. *Plant J. Cell Mol. Biol.* 41 710–721. 10.1111/j.1365-313X.2004.02330.x 15703058

[B18] Kim W. C. Kim J. Y. Ko J. H. Kang H. Han K. H. (2014a). Identification of direct targets of transcription factor MYB46 provides insights into the transcriptional regulation of secondary wall biosynthesis. *Plant Mol. Biol.* 85 589–599. 10.1007/s11103-014-0205-x 24879533

[B19] Kim W. C. Reca I. B. Kim Y. S. Park S. Thomashow M. F. Keegstra K. (2014b). Transcription factors that directly regulate the expression of CSLA9 encoding mannan synthase in *Arabidopsis thaliana*. *Plant Mol. Biol.* 84 577–587. 10.1007/s11103-013-0154-9 24243147

[B20] Kim W. C. Ko J. H. Kim J. Y. Kim J. Bae H. J. Han K. H. (2013). MYB46 directly regulates the gene expression of secondary wall-associated cellulose synthases in Arabidopsis. *Plant J.* 73 26–36. 10.1111/j.1365-313x.2012.05124.x 26011122

[B21] Lacombe B. Achard P. (2016). Long-distance transport of phytohormones through the plant vascular system. *Curr. Opin. Plant Biol.* 34 1–8. 10.1016/j.pbi.2016.06.007 27340874

[B22] Li J. Wang X. Cui Y. (2014). Uncovering the overlapping community structure of complex networks by maximal cliques. *Physica A* 415 398–406. 10.1016/j.physa.2014.08.025

[B23] Li X. P. Björkman O. Shih C. Grossman A. R. Rosenquist M. Jansson S. (2000). A pigment-binding protein essential for regulation of photosyntheticlight harvesting. *Nature* 403 391–395. 10.1038/35000131 10667783

[B24] Li Y. Pearl S. A. Jackson S. A. (2015). Gene networks in plant biology: approaches in reconstruction and analysis. *Trends Plant Sci.* 20 664–675. 10.1016/j.tplants.2015.06.013 26440435

[B25] Mccarthy R. L. Zhong R. Ye Z. H. (2009). MYB83 is a direct target of SND1 and acts redundantly with MYB46 in the regulation of secondary cell wall biosynthesis in arabidopsis. *Plant Cell Physiol.* 50 1950–1964. 10.1093/pcp/pcp139 19808805

[B26] Montané M. H. Kloppstech K. (2000). The family of light-harvesting-related proteins (LHCs, ELIPs, HLIPs): was the harvesting of light their primary function? *Gene* 258 1–8. 10.1016/S0378-1119(00)00413-3 11111037

[B27] Morenorisueno M. A. Busch W. Benfey P. N. (2010). Omics meet networks - using systems approaches to infer regulatory networks in plants. *Curr. Opin. Plant Biol.* 13 126–131. 10.1016/j.pbi.2009.11.005 20036612 PMC2862083

[B28] Ohnishi T. Godza B. Watanabe B. Fujioka S. Hategan L. Ide K. (2012). CYP90A1/CPD, a brassinosteroid biosynthetic cytochrome P450 of arabidopsis, catalyzes C-3 oxidation. *J. Biol. Chem.* 287 31551–31560. 10.1074/jbc.M112.392720 22822057 PMC3438987

[B29] Peng Z. Lu Y. Li L. Zhao Q. Feng Q. Gao Z. (2013a). The draft genome of the fast-growing non-timber forest species moso bamboo (*Phyllostachys heterocycla*). *Nat. Genet.* 45 456–461. 10.1038/ng.2569 23435089

[B30] Peng Z. Zhang C. Zhang Y. Hu T. Mu S. Li X. (2013b). Transcriptome sequencing and analysis of the fast growing shoots of moso bamboo (*Phyllostachys edulis*). *PLoS One* 8:e78944. 10.1371/journal.pone.0078944 24244391 PMC3820679

[B31] Serin E. A. R. Harm N. Hilhorst H. W. M. Wilco L. (2016). Learning from co-expression networks: possibilities and challenges. *Front. Plant Sci.* 7:444. 10.3389/fpls.2016.00444 27092161 PMC4825623

[B32] Speir M. L. Zweig A. S. Rosenbloom K. R. Raney B. J. Paten B. Nejad P. (2016). The UCSC genome browser database: 2016 update. *Nucleic Acids Res.* 44 D717–D725. 10.1093/nar/gkv1275 26590259 PMC4702902

[B33] Tian T. Liu Y. Yan H. You Q. Yi X. Du Z. (2017). agriGO v2.0: a GO analysis toolkit for the agricultural community, 2017 update. *Nucleic Acids Res.* 45 W122–W129. 10.1093/nar/gkx382 28472432 PMC5793732

[B34] Trapnell C. Pachter L. Salzberg S. L. (2009). TopHat: discovering splice junctions with RNA-Seq. *Bioinformatics* 25 1105–1111. 10.1093/bioinformatics/btp120 19289445 PMC2672628

[B35] Trapnell C. Williams B. A. Pertea G. Mortazavi A. Kwan G. Van Baren M. J. (2010). Transcript assembly and quantification by RNA-Seq reveals unannotated transcripts and isoform switching during cell differentiation. *Nat. Biotechnol.* 28 511–515. 10.1038/nbt.1621 20436464 PMC3146043

[B36] Usadel B. Obayashi T. Mutwil M. Giorgi F. M. Bassel G. W. Tanimoto M. (2009). Co-expression tools for plant biology: opportunities for hypothesis generation and caveats. *Plant Cell Environ.* 32 1633–1651. 10.1111/j.1365-3040.2009.02040.x 19712066

[B37] Wei Q. Jiao C. Guo L. Ding Y. Cao J. Feng J. (2016). Exploring key cellular processes and candidate genes regulating the primary thickening growth of Moso underground shoots. *New Phytol.* 214 81–96. 10.1111/nph.14284 27859288

[B38] Xu P. Mohorianu I. Yang L. Zhao H. Gao Z. Dalmay T. (2014). Small RNA profile in moso bamboo root and leaf obtained by high definition adapters. *PLoS One* 9:e103590. 10.1371/journal.pone.0103590 25079776 PMC4117519

[B39] Yi X. Du Z. Su Z. (2013). PlantGSEA: a gene set enrichment analysis toolkit for plant community. *Nucleic Acids Res.* 41 W98–W103. 10.1093/nar/gkt281 23632162 PMC3692080

[B40] Yi Z. Chen J. Sun H. Rosli H. G. Pombo M. A. Zhang P. (2016). iTAK: a program for genome-wide prediction and classification of plant transcription factors,transcriptional regulators, and protein kinases. *Mol. Plant* 9 1667–1670. 10.1016/j.molp.2016.09.014 27717919

[B41] You Q. Xu W. Zhang K. Zhang L. Yi X. Yao D. (2017). ccNET: database of co-expression networks with functional modules for diploid and polyploid *Gossypium*. *Nucleic Acids Res.* 45 D1090–D1099. 10.1093/nar/gkw910 28053168 PMC5210623

[B42] You Q. Zhang L. Yi X. Zhang K. Yao D. Zhang X. (2016). Co-expression network analyses identify functional modules associated with development and stress response in *Gossypium arboreum*. *Sci. Rep.* 6:38436. 10.1038/srep38436 27922095 PMC5138846

[B43] Yu J. Zhang Z. Wei J. Yi L. Xu W. Su Z. (2014). SFGD: a comprehensive platform for mining functional information from soybean transcriptome data and its use in identifying acyl-lipid metabolism pathways. *BMC Genomics* 15:271. 10.1186/1471-2164-15-271 24712981 PMC4051163

[B44] Yukihisa S. Hideki G. Ayako N. Suguru T. Shozo F. Shigeo Y. (2003). Organ-specific expression of brassinosteroid-biosynthetic genes and distribution of endogenous brassinosteroids in Arabidopsis. *Plant Physiol.* 131 287–297. 10.1104/pp.013029 12529536 PMC166808

[B45] Zhao H. Gao Z. Wang L. Wang J. Wang S. Fei B. (2018). Chromosome-level reference genome and alternative splicing atlas of moso bamboo (*Phyllostachys edulis*). *Gigascience* 7:giy115. 10.1093/gigascience/giy115 30202850 PMC6204424

[B46] Zhao H. Lou Y. Sun H. Li L. Wang L. Dong L. (2016). Transcriptome and comparative gene expression analysis of *Phyllostachys edulis* in response to high light. *BMC Plant Biol.* 16:34. 10.1186/s12870-016-0720-9 26822690 PMC4730629

[B47] Zhao H. Peng Z. Fei B. Li L. Hu T. Gao Z. (2014). BambooGDB: a bamboo genome database with functional annotation and an analysis platform. *Database* 2014:bau006. 10.1093/database/bau006 24602877 PMC3944406

[B48] Zhao H. Zhao S. International Network for Bamboo and Rattan Fei B. Liu H. Yang H. (2017). Announcing the genome atlas of bamboo and rattan (GABR) project: promoting research in evolution and in economically and ecologically beneficial plants. *Gigascience* 6 1–7. 10.1093/gigascience/gix046 28637269 PMC5570132

[B49] Zhou J. Lee C. Zhong R. Ye Z. H. (2009). MYB58 and MYB63 are transcriptional activators of the lignin biosynthetic pathway during secondary cell wall formation in Arabidopsis. *Plant Cell* 21 248–266. 10.1105/tpc.108.063321 19122102 PMC2648072

