# Supplementary material for: Co-expression Gene Network Analysis and Functional Module Identification in Bamboo Growth and Development
Source: Front Genet. 2018 Nov 27;9:574. doi: 10.3389/fgene.2018.00574 (PMC6277748; doi:10.3389/fgene.2018.00574)
Supplement: Supplementary file 1 [file Data_Sheet_1.pdf]

Supplementary Material

Co-expression Gene Network Analysis and Functional Module Identification in Bamboo Growth and Development

Xuelian Ma[1†], Hansheng Zhao[2†], Wenying Xu[1], Qi You[1], Hengyu Yan[1], Zhimin Gao[2]*, Zhen Su[1]*

[†]These authors have contributed equally to this work.

*To whom correspondence should be addressed: Zhen Su (zhensu@cau.edu.cn); Zhimin Gao (gaozhimin@icbr.ac.cn)

Supplementary Figures

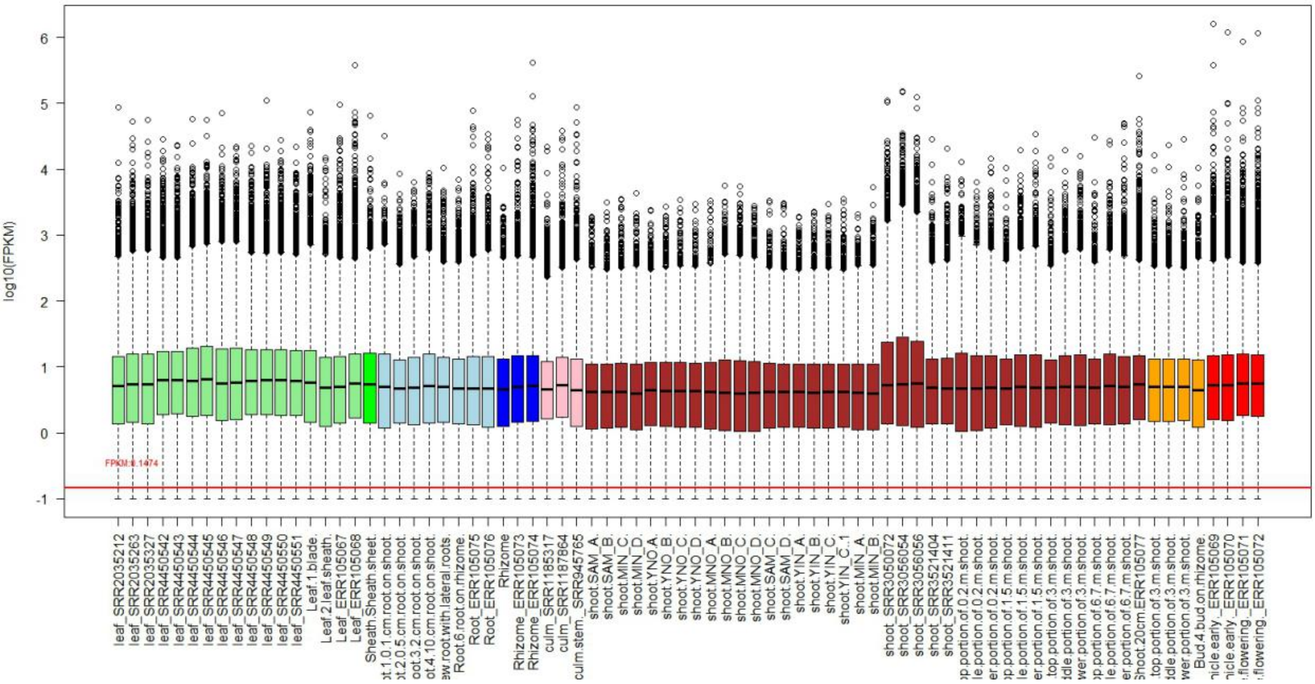

Supplementary Figure 1. The distribution of all genes' FPKM values

The boxplot displays the distribution of all genes' FPKM values among the 78 different samples. The x-axis lists the 78 RNA-seq samples and the y-axis is the gene expression value ($\log_{10} FPKM$). Here, the red horizontal line represents the threshold for the minimum FPKM (0.1474) of the expressed gene.

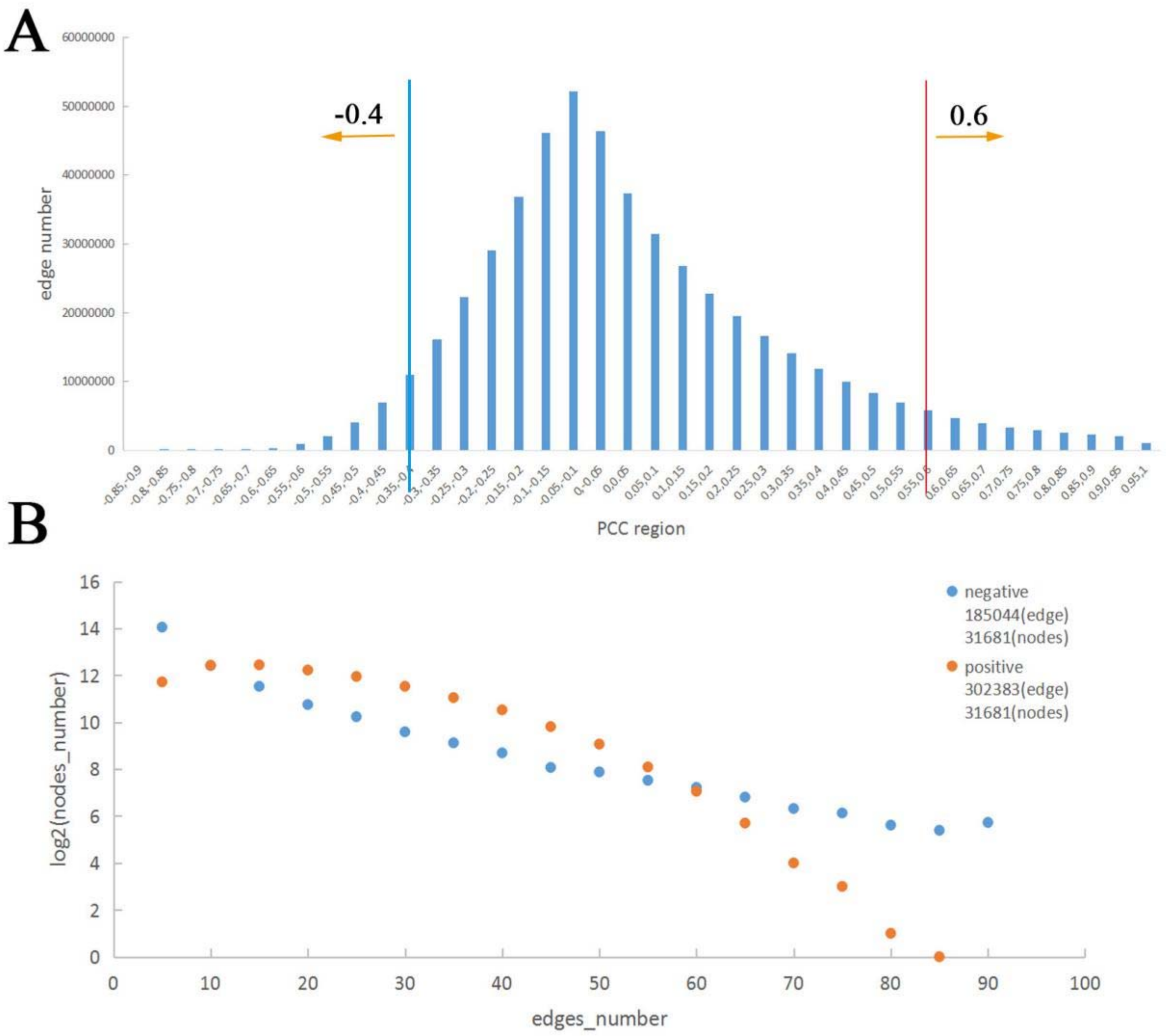

Supplementary Figure 2. Co-expression network construction parameter

(A) The PCC distribution of all gene pairs in bamboo. The lowest 5% and highest 5% of PCC values, namely, -0.4 and 0.6, were set as thresholds, and gene pairs with PCC values in the relevant region were regarded as co-expressed. **(B)** The scatter plot contains the statistical results for the nodes and edges of the MR co-expression network. The y-axis represents nodes and the x-axis represents edges in the MR network. An orange dot indicates that there are y nodes with x edges in the positive co-expression network (PCC > 0). A blue dot indicates that there are y nodes with x edges in the negative co-expression network (PCC < 0).

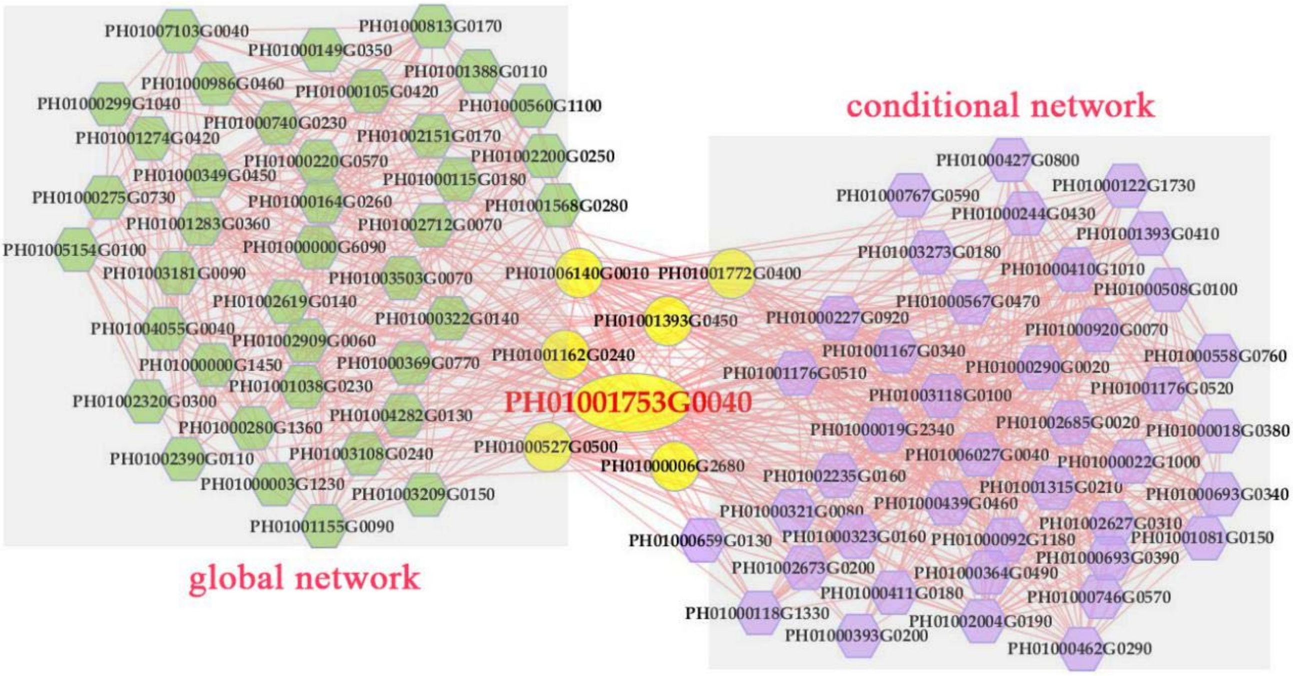

Supplementary Figure 3. Comparison of the global network and the conditional network

An example of a comparison result between the global and conditional co-expression networks. The yellow nodes stand for overlaps of the two networks, the light-green nodes stand for unique genes in the global sub-network, and the purple nodes stand for unique genes in conditional sub-network. A light-red line connects two genes with positive co-expression relationships.

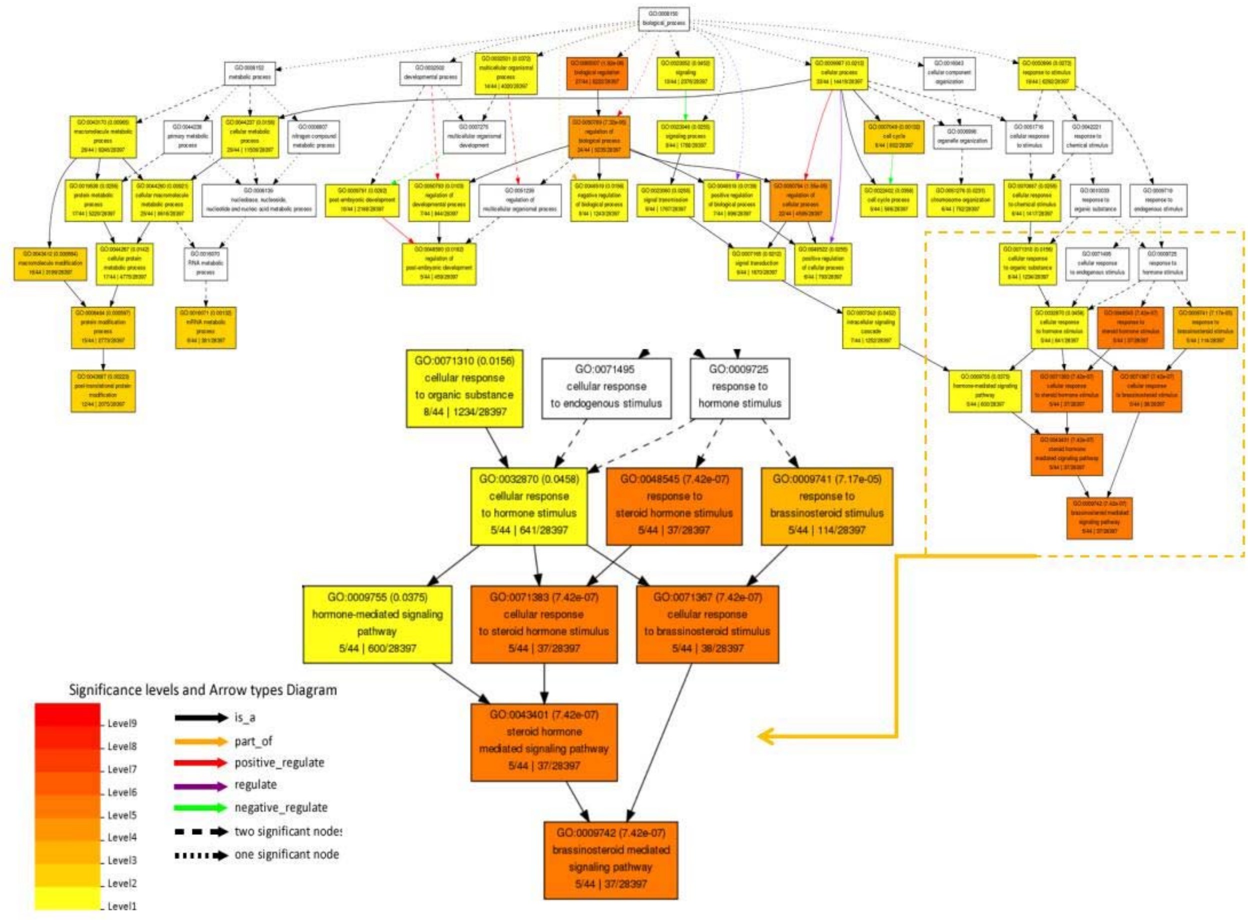

Supplementary Figure 4. GO enrichment analysis of co-expressed genes from the global network related to BR signal transduction genes *BAK1* (PH01000234G0890) and *BIN2* (PH01000584G0630) in bamboo

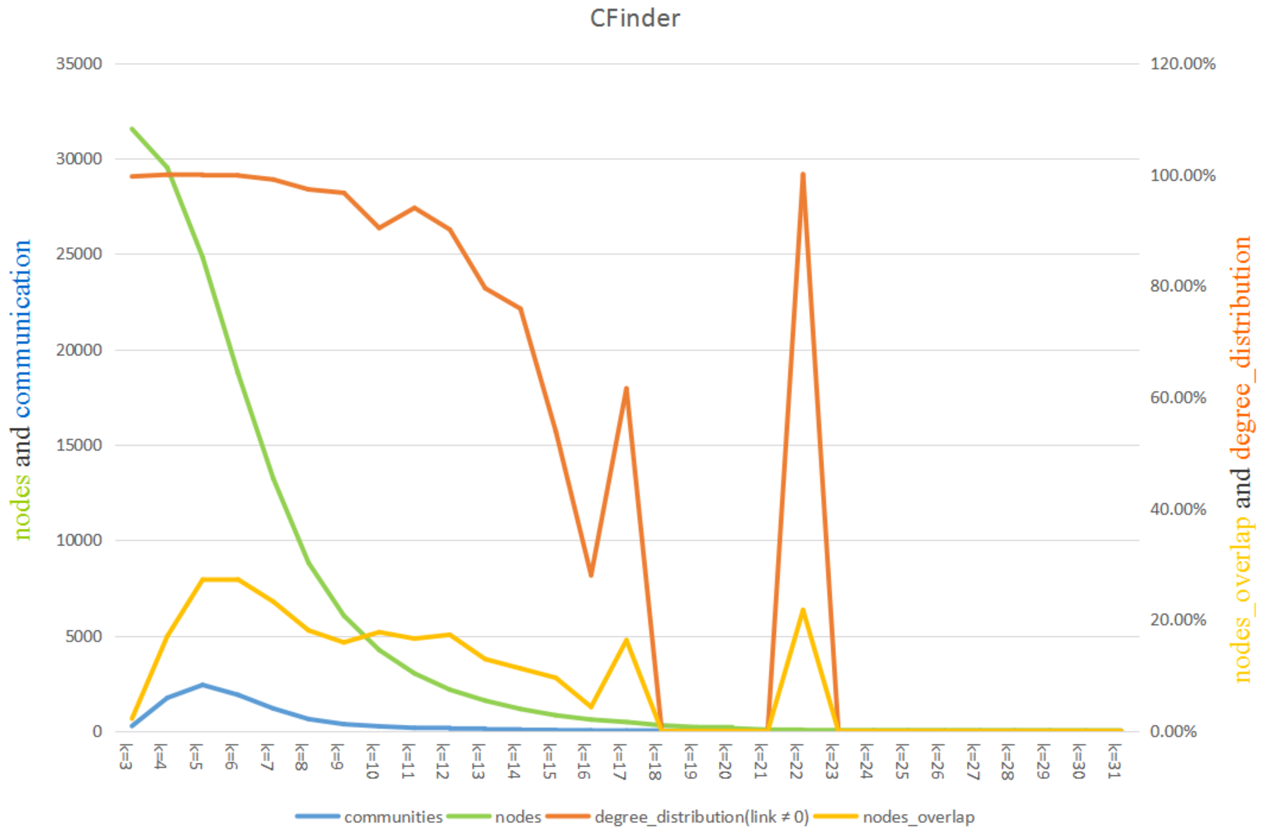

Supplementary Figure 5. Proper selection for function module size

The tool CFinder was applied to calculate communities of different k-clique sizes (from k = 3 to k = 31). Statistics of the communities of different k-clique sizes, including community number, community degree, community-community overlap and community total node were compared. Here, community number represents the number of communities of a selected k-clique size; community degree represents the number of other communities overlapping with a selected community; community-community overlap represents the number of nodes contained by two overlapping communities; community total nodes represents the total genes contained in a given k-clique size. The left y-axis represents the community number and community total nodes, while the right y-axis represents the community degree ratio (number of communities with overlapped communities/number of total communities) and community-community overlap ratio (number of nodes contained by two overlapping communities/community total node). The clique size k = 6 contains the most communities, the highest community-community overlap ratio and the top community degree ratio. We selected the k = 6 clique because it offered more possible functional modules (communities), more gene coverage and more community overlap (mimicking crosslinks of biological processes).
