# Supplementary material for: Co-expression Gene Network Analysis and Functional Module Identification in Bamboo Growth and Development
Source: Front Genet. 2018 Nov 27;9:574. doi: 10.3389/fgene.2018.00574 (PMC6277748; doi:10.3389/fgene.2018.00574)
Supplement: Supplementary file 2 [file Data_Sheet_2.PDF]

Table S1. The details of mapping results in RNA-seq samples for global co-expression network construction

Samples	left (single)			right			overall
	Input	Mapped	Mapped rate of input	Input	Mapped	Mapped rate of input	
SRR2035212	40554208	30838956	76.00%	40554208	29285166	72.20%	74.10%
SRR2035263	48689134	37339811	76.70%	48689134	35483124	72.90%	74.80%
SRR2035327	72133675	55018594	76.30%	72133675	52146644	72.30%	74.30%
SRR4450542	20814096	14792250	71.10%	20814096	13932353	66.90%	69.00%
SRR4450543	20861739	14688387	70.40%	20861739	13723944	65.80%	68.10%
SRR4450544	15753907	10828440	68.70%	15753907	10460254	66.40%	67.60%
SRR4450545	15872147	11341157	71.50%	15872147	10768290	67.80%	69.60%
SRR4450546	19291320	13565975	70.30%	19291320	12921198	67.00%	68.70%
SRR4450547	23557419	16443740	69.80%	23557419	15478399	65.70%	67.80%
SRR4450548	27484397	19601675	71.30%	27484397	18462614	67.20%	69.20%
SRR4450549	20035229	14248563	71.10%	20035229	13420396	67.00%	69.10%
SRR4450550	21494230	15144312	70.50%	21494230	14444593	67.20%	68.80%
SRR4450551	20785835	14496863	69.70%	20785835	13735638	66.10%	67.90%
SRR945765	22931972	13801467	60.20%	22931972	14911307	65.00%	62.60%
SRR4209871	8595961	7668666	89.20%	8595961	7950411	92.50%	90.90%
SRR4209872	8433639	6902989	81.90%	8433639	7838439	92.90%	87.40%
SRR4209873	7836014	7182902	91.70%	7836014	7323964	93.50%	92.60%
SRR4209874	7007869	6389976	91.20%	7007869	6518125	93.00%	92.10%
SRR4209875	8192694	7394233	90.30%	8192694	7578591	92.50%	91.40%
SRR4209876	5281289	4075451	77.20%	5281289	4939746	93.50%	85.40%
SRR4209877	8274420	7480480	90.40%	8274420	7645446	92.40%	91.40%
SRR4209878	8153619	7332191	89.90%	8153619	7536256	92.40%	91.20%
SRR4209879	6304275	5641962	89.50%	6304275	5811016	92.20%	90.80%
SRR4209880	6318686	5692817	90.10%	6318686	5850934	92.60%	91.30%
SRR4209881	7447159	6765111	90.80%	7447159	6913534	92.80%	91.80%
SRR4209882	7301646	6647653	91.00%	7301646	6772215	92.70%	91.90%
SRR4209883	7585829	6838910	90.20%	7585829	7001120	92.30%	91.20%

SRR4209884	7332748	6718828	91.60%	7332748	6861429	93.60%	92.60%
SRR4209885	7790328	6855990	88.00%	7790328	7207712	92.50%	90.30%
SRR4209886	8846602	7842816	88.70%	8846602	8162948	92.30%	90.50%
SRR4209887	7353768	6585765	89.60%	7353768	6769320	92.10%	90.80%
SRR4209888	7052260	6389625	90.60%	7052260	6559256	93.00%	91.80%
SRR4209889	7059326	6330859	89.70%	7059326	6506207	92.20%	90.90%
SRR4209890	7220549	6388339	88.50%	7220549	6611160	91.60%	90.00%
SRR3050072	65213884	52431356	80.40%	65213884	51434493	78.90%	79.60%
SRR3056054	89673012	72296161	80.60%	89673012	71051424	79.20%	79.90%
SRR3056056	94535103	75665466	80.00%	94535103	74301725	78.60%	79.30%
SRR3521404	148511051	101336801	68.20%	148511051	95774358	64.50%	66.40%
SRR3521411	140218515	95989658	68.50%	140218515	89747579	64.00%	66.20%
ERR105067	22661499	10828366	47.80%	22661499	7096840	31.30%	39.50%
ERR105068	18743096	12952879	69.10%	18743096	12730275	67.90%	68.50%
ERR105069	26665154	17140873	64.30%	26665154	11048676	41.40%	52.90%
ERR105070	21649475	10732530	49.60%	21649475	6275464	29.00%	39.30%
ERR105071	12521729	7545139	60.30%	12521729	4097042	32.70%	46.50%
ERR105072	17597114	12758540	72.50%	17597114	11940889	67.90%	70.20%
ERR105073	23710288	11459229	48.30%	23710288	6910258	29.10%	38.70%
ERR105074	16990412	11556329	68.00%	16990412	11286710	66.40%	67.20%
ERR105075	24882144	17547978	70.50%	24882144	10707946	43.00%	56.80%
ERR105076	24362472	11819414	48.50%	24362472	7885011	32.40%	40.40%
ERR105077	156395970	136074980	87.00%	156395970	131931863	84.40%	85.70%
SRR1185317	44016385	38052163	86.40%				
SRR1187864	67961501	55866472	82.20%	67961501	55457559	81.60%	81.90%
Leaf-1(blade)	21518564	14333097	66.60%	21518564	13336934	62.00%	64.30%
	22074233	14631030	66.30%	22074233	13547445	61.40%	63.80%
Leaf-2(leaf sheath)	23031696	14545377	63.20%	23031696	13553921	58.80%	61.00%
	23530647	14784566	62.80%	23530647	13726269	58.30%	60.60%
Sheath(Sheath sheet)	27584852	18883389	68.50%	27584852	17579291	63.70%	66.10%

Sheath(Sheath sheet)	28034884	19107694	68.20%	28034884	17703054	63.10%	65.70%
Root-1(0.1 cm root on shoot)	20168112	13219680	65.50%	20168112	12499831	62.00%	63.80%
	19735806	12678635	64.20%	19735806	12064679	61.10%	62.70%
Root-2(0.5 cm root on shoot)	25991515	17716977	68.20%	25991515	16498529	63.50%	65.80%
	25814848	17236061	66.80%	25814848	16156924	62.60%	64.70%
Root-3(2 cm root on shoot)	23259860	16394783	70.50%	23259860	15222607	65.40%	68.00%
	23725174	16659547	70.20%	23725174	15384682	64.80%	67.50%
Root-4(10 cm root on shoot)	23161324	15606886	67.40%	23161324	14747999	63.70%	65.50%
	22916628	15126070	66.00%	22916628	14377571	62.70%	64.40%
Root-5(new root with lateral roots)	22899763	15072995	65.80%	22899763	14347896	62.70%	64.20%
	22483533	14510151	64.50%	22483533	13903539	61.80%	63.20%
Root-6(root on rhizome)	22504135	15208431	67.60%	22504135	14263880	63.40%	65.50%
	22048062	14593500	66.20%	22048062	13782656	62.50%	64.40%
Rhizome	25571354	17403872	68.10%	25571354	15995533	62.60%	65.30%
	25200240	16800666	66.70%	25200240	15544456	61.70%	64.20%
Shoot-A1(top portion of 0.2 m shoot)	25241767	17261302	68.40%	25241767	16249296	64.40%	66.40%
	24949303	16700515	66.90%	24949303	15863000	63.60%	65.30%
Shoot-A2(middle portion of 0.2 m shoot)	21942435	11951303	54.50%	21942435	13153055	59.90%	57.20%
	22450363	11984642	53.40%	22450363	13330915	59.40%	56.40%
Shoot-A3(lower portion of 0.2 m shoot)	25762804	17368147	67.40%	25762804	16349607	63.50%	65.40%
	25405546	16778146	66.00%	25405546	15893883	62.60%	64.30%
Shoot-B1(top portion of 1.5 m shoot)	19780898	9222425	46.60%	19780898	11402898	57.60%	52.10%
	19353839	9680329	50.00%	19353839	11049154	57.10%	53.60%
Shoot-B2(middle portion of 1.5 m shoot)	30357134	20482547	67.50%	30357134	18972916	62.50%	65.00%
	29759839	19671124	66.10%	29759839	18372028	61.70%	63.90%
Shoot-B3(lower portion of 1.5 m shoot)	24433260	16324714	66.80%	24433260	15040060	61.60%	64.20%
	23736142	15540136	65.50%	23736142	14408216	60.70%	63.10%
Shoot-C1(top portion of 3 m shoot)	21188480	14154098	66.80%	21188480	13253052	62.50%	64.70%
	21668124	14416344	66.50%	21668124	13443089	62.00%	64.30%
Shoot-C2(middle portion of 3 m shoot)	24017812	16682684	69.50%	24017812	15585860	64.90%	67.20%

Shoot-C2(middle portion of 3 m shoot)	24471985	16927455	69.20%	24471985	15743324	64.30%	66.80%
Shoot-C3(lower portion of 3 m shoot)	27820171	18918053	68.00%	27820171	17839288	64.10%	66.10%
	27876598	18566966	66.60%	27876598	17632343	63.30%	64.90%
Shoot-D1(top portion of 6.7 m shoot)	24247093	15987378	65.90%	24247093	15275419	63.00%	64.50%
	24488351	16086708	65.70%	24488351	15316584	62.50%	64.10%
Shoot-D2(middle portion of 6.7 m shoot)	20506009	9732502	47.50%	20506009	12758288	62.20%	54.80%
	20886051	9438260	45.20%	20886051	12864129	61.60%	53.40%
Shoot-D3(lower portion of 6.7 m shoot)	29514308	19549502	66.20%	29514308	18374935	62.30%	64.20%
	29797369	19672248	66.00%	29797369	18407735	61.80%	63.90%
Bud-1(bud on top portion of 3 m shoot)	27142154	18171056	66.90%	27142154	16863283	62.10%	64.50%
	27860357	18558714	66.60%	27860357	17154970	61.60%	64.10%
Bud-2(bud on middle portion of 3 m shoot)	23029769	15268855	66.30%	23029769	14408746	62.60%	64.40%
	23430114	15467131	66.00%	23430114	14542861	62.10%	64.00%
Bud-3(bud on lower portion of 3 m shoot)	24767795	16683578	67.40%	24767795	15494903	62.60%	65.00%
	25525945	17115657	67.10%	25525945	15833810	62.00%	64.50%
Bud-4(bud on rhizome)	25015665	16613964	66.40%	25015665	15370018	61.40%	63.90%
	24436061	15902738	65.10%	24436061	14827873	60.70%	62.90%
